# Eosinophilic Esophagitis and Autoimmune Thyroid Disease: A Population‐Based Matched Cohort Study

**DOI:** 10.1002/ueg2.70248

**Published:** 2026-07-01

**Authors:** Soran R. Bozorg, David Bergman, Fahim Ebrahimi, Bjorn Roelstraete, Marie Carlson, Amiko M. Uchida, Evan S. Dellon, Jonas F. Ludvigsson

**Affiliations:** ^1^ Department of Medical Epidemiology and Biostatistics Karolinska Institutet Solna Sweden; ^2^ Division of Gastroenterology Karolinska University Hospital Solna Sweden; ^3^ Department of Gastroenterology Clarunis University Centre for Gastrointestinal and Liver Disease Basel Switzerland; ^4^ Department of Medical Sciences Uppsala University Uppsala Sweden; ^5^ Division of Gastroenterology, Hepatology, and Nutrition University of Utah School of Medicine Salt Lake City Utah USA; ^6^ Department of Medicine University of North Carolina School of Medicine Chapel Hill North Carolina USA; ^7^ Department of Pediatrics Örebro University Hospital Örebro Sweden; ^8^ Department of Medicine Columbia University Medical Center Columbia New York USA

**Keywords:** autoimmune comorbidity, autoimmune thyroid disease, dysphagia, eosinophilic esophagitis, esophageal disease

## Abstract

**Background:**

Eosinophilic esophagitis (EoE) is an immune‐mediated disease typically presenting with esophageal symptoms. Although prior exploratory studies on the autoimmune comorbidity of EoE have indicated a possible association with autoimmune thyroid disease (AITD), this relationship has not been examined in detail.

**Methods:**

Using the nationwide ESPRESSO cohort consisting of data from all 28 pathology departments in Sweden, we identified patients with biopsy‐confirmed EoE diagnosed between 2006–2023. Each EoE patient was compared with general‐population references matched on sex, age, county of residence, and calendar year of diagnosis. A secondary reference group consisted of full siblings of EoE patients. Cox proportional hazard model was used to estimate the hazard ratio (HR) of future AITD, whereas a logistic regression model was used to estimate the odds ratio (OR) of prior AITD.

**Results:**

During follow‐up, 70/5801 EoE patients developed AITD (incidence rate, 2.14/1000 person‐years) compared to 271/27,407 references (1.74/1000 person‐years), corresponding to an HR of 1.18 (95% CI, 0.91–1.54). Compared to sibling comparators, the HR was 1.27 (0.84–1.94). The prevalence of *prior* AITD was 3.9% in EoE, compared with 3.0% in references, corresponding to an OR of 1.29 (1.10–1.49). Notably, in childhood‐onset EoE, the HR of *future* AITD was 2.53 (1.18–5.45) and the OR of *prior* AITD was 4.71 (2.34–9.43).

**Conclusions:**

EoE was associated with *prior* AITD, but not with *future* AITD. Childhood‐onset EoE was however associated with both *prior* and *future* AITD. Physicians diagnosing EoE should be observant of symptoms indicative of AITD, especially in childhood‐onset EoE.

## Key Summary

- Summarize the established knowledge on this subject:
  ◦ Eosinophilic esophagitis (EoE) is an immune‐mediated disease typically presenting with esophageal symptoms.
  ◦ While prior exploratory studies have indicated a possible association between EoE and autoimmune thyroid disease (AITD), this relationship has not been examined in detail.
- What are the significant and/or new findings of this study?
  ◦ EoE was associated with *prior* AITD, but not with *future* AITD.
  ◦ Notably, the association between EoE and AITD was driven by childhood‐onset EoE, which had a pronounced *bidirectional* association with AITD.
  ◦ Given the increased coexistence of AITD in patients with EoE, physicians diagnosing EoE should be observant of symptoms indicative of AITD.

## Graphical abstract

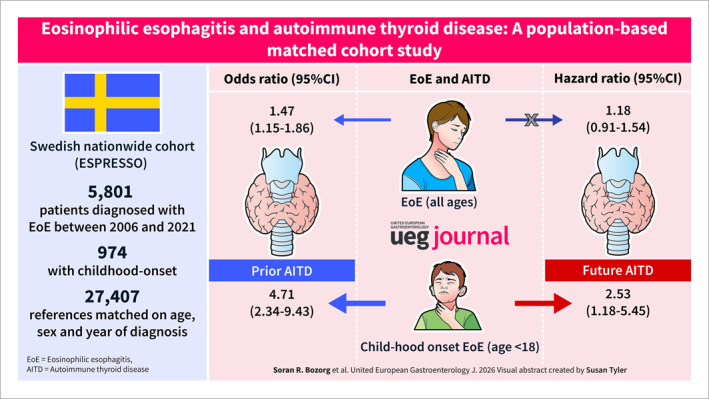

**Abbreviations**
AITD: Autoimmune Thyroid Disease
ATC: Anatomical Therapeutic Chemical
CI: Confidence Interval
EoE: Eosinophilic Esophagitis
HR: Hazard Ratio
ICD: International Classification of Diseases
OR: Odds Ratio
PY: Person‐years

## 1 Introduction

Eosinophilic esophagitis (EoE) is a chronic immune‐mediated disease characterized by an infiltration of eosinophils in the esophagus [1]. Disease onset typically occurs in children and young adults, and recent research has repeatedly demonstrated a rising incidence globally [2]. The clinical presentation of EoE relates to the esophageal inflammation which causes dysphagia [3]. However, in addition to symptoms consistent with EoE, the diagnostic criteria also require histopathology to confirm an increased eosinophilic count in the esophageal mucosa [4, 5].

Associated comorbidities are known to contribute to the disease burden in EoE and may have significant implications on quality‐of‐life. In comparison to other comparable diseases, less is known about the immune‐mediated comorbidity of EoE. It has been suggested that EoE is part of the atopic march due to its association with other allergic conditions, including asthma, eczema, and allergic rhinitis [6, 7]. Furthermore, previous exploratory studies have identified associations with other autoimmune diseases, including autoimmune thyroid disease (AITD) [8]. In a retrospective study based on the UNC Clinicopathologic EoE cohort (*n* = 1029), Hashimoto's disease was found to be the second most common non‐atopic autoimmune condition among newly diagnosed EoE patients with a prevalence of 1.2% [9]. Another study, based on the Utah Population Database (*n* > 4300), found that the prevalence of Hashimoto's disease among patients with EoE was 0.8%, and that patients with EoE had an increased risk of prior AITD compared with age‐ and sex‐matched controls (odds ratio [OR], 3.09) [10].

Although these initial studies on autoimmune conditions in general may indicate an association between EoE and AITD, no prior studies have examined the association between EoE and AITD in detail or elucidated their temporal relationship.

## 2 Methods

In this large nationwide study, a matched cohort design was used to estimate the risk of future AITD (as defined below) in patients with EoE. Furthermore, we used a case‐control design to evaluate the risk of prior AITD in patients with EoE.

### 2.1 Study Setting

This study was conducted in Sweden, which has a tax‐funded healthcare system with nearly universal access to healthcare and prescription drugs [11]. There are principally two levels of healthcare: primary healthcare and specialized healthcare. In Sweden, patients with EoE are exclusively managed within specialized healthcare. Regarding AITD, hyperthyroidism is generally initially managed within specialized healthcare, whereas uncomplicated hypothyroidism is primarily managed within primary healthcare.

### 2.2 Data Sources

Most of the data utilized in this study originates from nationwide health registers maintained by Swedish government agencies. The National Patient Register, which attained nationwide coverage of specialized inpatient care in 1987 and specialized outpatient care in 2001, stores data such as diagnostic codes (according to the International Classification of Diseases, ICD) and procedure codes recorded for each specific healthcare episode [12]. The Prescribed Drug Register collects data on all dispensed prescription medications, including Anatomic Therapeutic Chemical (ATC) codes, and has complete coverage since 2005 [13]. The Total Population Register maintains complete records on all Swedish residents since 1968, and a subset of the register called the Multigenerational Register also contains data on family relations (e.g., siblings, parents) for all Swedish residents [14]. Lastly, the Longitudinal Integrated database for health insurance and labor market studies (the LISA database) holds data on sociodemographic factors, including highest attained education level (< 2% missing data), which is commonly used as a proxy for socioeconomic status [15].

In addition to the public health registers, this study also leverages data from the ESPRESSO cohort (*Epidemiology Strengthened by histoPathology Reports in Sweden*) that covers all of Sweden's 28 pathology departments [16]. The cohort currently consists of all individuals in Sweden with at least one gastrointestinal biopsy between 1965 and 2023, which equals approximately 2.6 million unique individuals and more than 8 million data entries [17].

### 2.3 Study Population

Through the ESPRESSO cohort, we identified individuals with biopsy‐verified EoE diagnosed between 2006 and 2023. We chose 2006 as the starting point due to the requirement of data from the Prescribed Drug Register as part of our exposure and outcome definitions as described below. Exclusion criteria for the cohort analysis included a history of prior AITD, thyroid cancer, thyroid gland procedure, or use of iodine‐containing medications *before* the index date/date of diagnosis. For the case‐control analysis, we excluded prior thyroid cancer or use of iodine‐containing medications, or thyroid gland procedures when they occurred more than 1 year before AITD diagnosis (Supporting Information S1: Figure S1).

Each patient with EoE was matched with up to five reference individuals from the general population based on age, sex, county of residence, and calendar year of diagnosis (date of biopsy). We also identified a second reference group consisting of full siblings without EoE or AITD. This reference group was used as a means to examine the potential role of shared genetics and early‐life environmental factors.

### 2.4 Exposure and Outcome Definitions

A diagnosis of EoE was considered to be the exposure in the cohort analysis, and an outcome in the case‐control analysis, and it could be ascertained in two ways. Namely, EoE was defined as either having (1) a biopsy report with topography code T62 (esophagus) and SNOMED code M47150 (*eosinophilic inflammation*), or (2) a biopsy report with topography code T62 and SNOMED code M4* (*inflammation*) combined with a record of ICD code K20.9 (*eosinophilic esophagitis*) in the National Patient Register. Codes related to the biopsy report are assigned by the pathologist as part of the histopathological assessment, and codes registered in the National Patient Register are assigned by the treating physician. Since the ICD code for EoE was introduced in Sweden 2012, the latter method could only be used to identify patients diagnosed with EoE 2012 or later. Of note, the presence of an esophageal biopsy with SNOMED code M47150 has previously been validated and found to have a positive predictive value of 89% for clinical EoE [18], and the combination of a consistent SNOMED code and ICD code has recently been used in another Swedish incidence study on EoE [19].

The index date was set to the date of biopsy for patients with EoE diagnosed solely based on histopathology, and the date for fulfilling both criteria for patients diagnosed with a combination of histopathology and ICD record.

AITD encompassed both autoimmune hyperthyroidism (e.g., Grave's disease) and autoimmune hypothyroidism (e.g., Hashimoto's disease). Congenital hypothyroidism, post‐infective thyroiditis, or pituitary dysfunction were not included in the definition, nor was post‐pregnancy thyroiditis. Ascertainment of AITD relied on the presence of relevant ICD codes recorded in the National Patient Register (Supporting Information S1: Table S1). In the absence of a previous record of autoimmune hyperthyroidism (Grave's disease), autoimmune hypothyroidism managed in primary healthcare could be ascertained through records of dispensed medications in the Prescribed Drug Register indicating the use of thyroid substitution hormones. This method has previously been used in other epidemiologic studies investigating AITD [20, 21].

### 2.5 Study Period and Follow‐Up

Follow‐up started in 2006 and continued through 2023. Follow‐up ended at the date of AITD occurrence, emigration, or death. Censoring was applied in the case of thyroid cancer or use of iodine‐containing medications before the outcome occurrence, or in the case of a thyroid gland procedure more than a year before the outcome occurrence. Reference individuals who developed EoE during follow‐up were censored and transferred to the exposure group and were equipped with their own set of reference individuals where they contributed time at risk from their time of EoE diagnosis.

### 2.6 Statistical Analysis

Relative risk estimates were reported as hazard ratios (HRs) with 95% confidence intervals (95% CIs) and were calculated using a Cox proportional hazards model. Furthermore, a Kaplan–Meier curve was used to illustrate cumulative incidence. We also reported incidence rates per 1000 person‐years (PY). Stratum‐specific analyses were performed by sex, age group (< 18, 18–39, 40–59, ≥ 60), years since diagnosis (< 1, 1–4, 5–9, ≥ 10), and country of birth (Nordic countries, other) as these variables are generally considered to be possible confounders. We also intended to examine the risk of hyper‐ and hypothyroidism separately, but abstained from this analysis since the number of individuals developing autoimmune hyperthyroidism was low.

To account for confounding due to sociodemographic factors, the baseline model was adjusted for the highest attained education level (≤ 9 years, 10–12 years, ≥ 13 years), as well as the matching variables (age, sex, county of residence, and calendar year of diagnosis) [22]. Additionally, a more comprehensive model was used to account for other possible causes of confounding: years since diagnosis (< 1, 1–4, 5–9, ≥ 10), calendar year period (−2011, 2012‐), country of birth (Nordic countries, other), baseline healthcare utilization (number of healthcare contacts 6–36 months before EoE diagnosis/index date: 0, 1–2, ≥ 3), baseline autoimmunity (binary variables for inflammatory bowel disease, celiac disease, and a composite measure of atopy consisting of asthma, allergic rhinitis, and atopic eczema), and chronic obstructive pulmonary disease (COPD; first diagnosed at age 40 or later as a proxy for smoking). The above specified covariates were carefully selected in order to avoid overfitting. Notably, covariates related to comorbidities were defined according to relevant ICD codes as registered in the National Patient Register (Supporting Information S1: Table S1). Moreover, a sibling‐controlled analysis was performed to account for potential confounding factors due to shared genetics and unmeasured early‐life factors.

Several pre‐specified sensitivity analyses were performed to test the robustness of the results. The case‐finding strategy of EoE was examined by (1) limiting the study population to those exclusively identified through a SNOMED code M47150, and (2) restricting the start of follow‐up to 2012; the year in which the updated EoE guidelines were published, which coincides with the year the ICD code for EoE was adopted in Sweden. The AITD definition was examined by restricting the outcome to those exclusively identified through ICD codes. Furthermore, potential surveillance bias was examined by starting follow‐up 1 year after the date of EoE diagnosis, and differences in healthcare seeking behavior were examined by excluding patients and references without any healthcare contacts 6–36 months before the index date.

In the case‐control analysis, we used a conditional logistic regression model to calculate ORs for prior AITD in patients diagnosed with EoE. The model was adjusted for the same set of covariates as in the cohort analysis (age, sex, county of residence, calendar year of diagnosis, and education level).

Data analyses were performed using R (version 3.6.0), primarily using the survival package, and a two‐sided *p* ≤ 0.05 was considered to be statistically significant.

### 2.7 Ethics

The study was approved by the Regional Ethical Review Board in Stockholm (reference number: 2014/1287–31/4, 2017/1497–32 and 2022–05774‐02) and informed consent was waived since the study was strictly register‐based.

## 3 Results

### 3.1 Patient Characteristics

We identified 6061 patients with incident EoE diagnosed between 2006 and 2023, of whom 5801 were included in the main analysis (Table 1) and 4253 had at least one full sibling comparator (Supporting Information S1: Table S2). Most EoE patients were male (75%), and the median age at the time of diagnosis was 40 (interquartile range, 25–55). Around 17% of the patients were diagnosed with EoE before the age of 18. The median follow‐up was 4.8 years (interquartile range, 2.8–8.1). As expected, atopy, inflammatory bowel disease, and celiac disease were more common in patients with EoE compared with the general population.

**TABLE 1:** Baseline characteristics of patients diagnosed with eosinophilic esophagitis (EoE) and general‐population comparators (reference).

	Reference^a^ (*n* = 27,407)	EoE (*n* = 5801)
	*n* [%]	*n* [%]
Sex		
Male	20,913 [76.31]	4353 [75.04]
Female	6494 [23.69]	1448 [24.96]
Age at start of follow‐up		
Mean [SD]	39.51 [19.75]	40.46 [20.03]
Median [IQR]	40.00 [24.00–54.00]	41.00 [25.00–55.00]
Range, min‐max	0–94	0–94
< 18	4821 [17.59]	974 [16.79]
18–39	8450 [30.83]	1732 [29.86]
40–59	9549 [34.84]	2029 [34.98]
≥ 60	4587 [16.74]	1066 [18.38]
Country of birth		
Nordic countries	22,081 [80.57]	5401 [93.10]
Other	5326 [19.43]	400 [6.90]
Highest attained education level		
Compulsory school (≤ 9 years)	4477 [16.34]	744 [12.83]
Upper secondary school (10–12 years)	9420 [34.37]	2024 [34.89]
College or university, (≥ 13 years)	7876 [28.74]	1961 [33.80]
Missing	5634 [20.56]	1072 [18.48]
Comorbidity at start of follow‐up		
Atopy^b^	2604 [9.50]	1564 [26.96]
Chronic pulmonary obstructive disease (COPD)^c^	178 [0.65]	61 [1.05]
Inflammatory bowel disease (IBD)	22 [0.08]	162 [2.79]
Celiac disease	13 [0.05]	127 [2.19]
Calendar year at start of follow‐up		
2006–2012	3030 [11.06]	625 [10.77]
2013–2017	8684 [31.69]	1818 [31.34]
2018–2023	15,693 [57.26]	3358 [57.89]
Years of follow‐up		
Mean [SD]	5.67 [3.68]	5.65 [3.68]
Median [IQR]	4.88 [2.77–8.16]	4.84 [2.76–8.12]
Range, min‐max	0.01–17.97	0.01–17.97
< 1	1672 [6.10]	340 [5.86]
1–4	12,395 [45.23]	2656 [45.79]
5–9	9394 [34.28]	1981 [34.15]
≥ 10	3946 [14.40]	824 [14.20]
Censoring events during follow‐up		
Thyroid cancer	7 [0.03]	1 [0.02]
Use of iodine‐containing drug	75 [0.28]	24 [0.42]
Thyroid gland procedure	0 [0.00]	0 [0.00]

Abbreviations: EoE, Eosinophilic esophagitis; IQR, Interquartile range; SD, Standard deviation.

^a^ General‐population references were matched on age, sex, county of residence, and calendar year of diagnosis.

^b^ Defined as asthma, allergic rhinitis, or atopic eczema (see Supporting Information S1: Table S1).

^c^ Restricted to those diagnosed at age 40 or later.

### 3.2 Risk of Future Autoimmune Thyroid Disease

**FIGURE 1:**
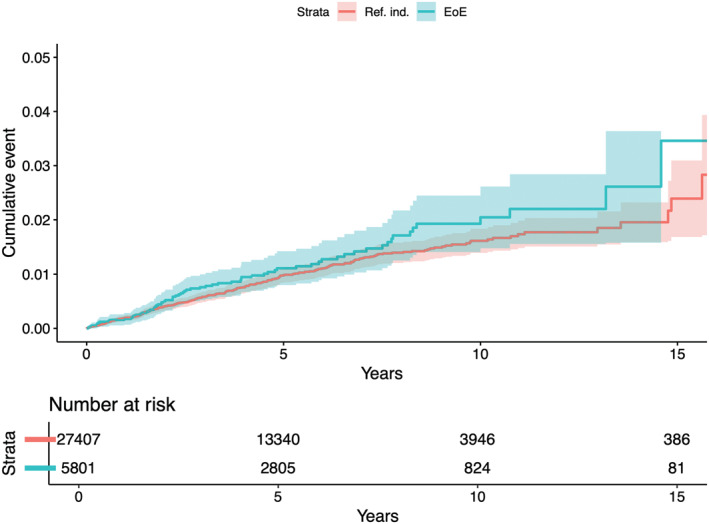
Kaplan‐Meier cumulative incidence curve of autoimmune thyroid disease (AITD) in patients diagnosed with eosinophilic esophagitis (EoE) and general‐population comparators (Ref. ind.).

During follow‐up, there were 70 AITD events (3 hyperthyroidism and 67 hypothyroidism) among the 5801 patients with EoE, as compared with 271 AITD events (12 hyperthyroidism and 259 hypothyroidism) among the 27,407 general‐population reference individuals (Figure 1). The corresponding incidence rates were 2.14/1000 PY and 1.74/1000 PY in patients with EoE and reference individuals, respectively, yielding an HR of 1.18 (95% CI, 0.91–1.54; *p* = 0.20; Table 2). Similar risk estimates were seen after additional adjustment in the more comprehensive model (HR, 1.20; 95% CI, 0.91–1.59). Using full siblings as the comparator, the HR for future AITD in patients with EoE was 1.27 (95% CI, 0.84–1.94; Table 3).

**TABLE 2:** Risk of autoimmune thyroid disease (AITD) in patient diagnosed with eosinophilic esophagitis (EoE) as compared with general‐population comparators (reference).

	Reference^a^ (*n* = 27,407)	EoE (*n* = 5801)
Number of events	271	70
Incidence rate per 1000 person‐years [95% CI]	1.74 [1.54–1.96]	2.14 [1.67–2.70]
Incidence rate difference [95% CI]	0 (ref)	0.39 [−0.15 to 0.94]
Hazard ratio, baseline model^b^	1 (ref)	1.18 [0.91–1.54]
Hazard ratio, comprehensive model^b^	1 (ref)	1.20 [0.91–1.59]

Abbreviations: CI, Confidence interval; EoE, Eosinophilic esophagitis.

^a^ General‐population references were matched on age, sex, county of residence, and calendar year of diagnosis.

^b^ The baseline model was adjusted for age, sex, county of residence, calendar year of diagnosis, and education level. The comprehensive model was additionally adjusted for years since diagnosis, calendar year period, country of birth, baseline healthcare utilization, baseline autoimmunity, and the presence of chronic pulmonary obstructive disease as a proxy for smoking.

**TABLE 3:** Risk of autoimmune thyroid disease (AITD) in patient diagnosed with eosinophilic esophagitis (EoE) as compared with sibling comparators (reference).

	Reference (*n* = 6791)	EoE (*n* = 4253)
Number of events	107	53
Incidence rate per 1000 person‐years [95% CI]	2.69 [2.21–3.25]	2.16 [1.61–2.82]
Incidence rate difference [95% CI]	0 (ref)	−0.54 [−1.31 to 0.24]
Hazard ratio, baseline model^a^	1 (ref)	1.27 [0.84–1.94]
Hazard ratio, comprehensive model^a^	1 (ref)	1.17 [0.75–1.83]

Abbreviations: CI, Confidence interval; EoE, Eosinophilic esophagitis.

^a^ The baseline model was adjusted for age, sex, county of residence, calendar year of diagnosis, and education level. The comprehensive model was additionally adjusted for years since diagnosis, calendar year period, country of birth, baseline healthcare utilization, baseline autoimmunity, and the presence of chronic pulmonary obstructive disease as a proxy for smoking.

#### 3.2.1 Stratum‐Specific Analyses

**FIGURE 2:**
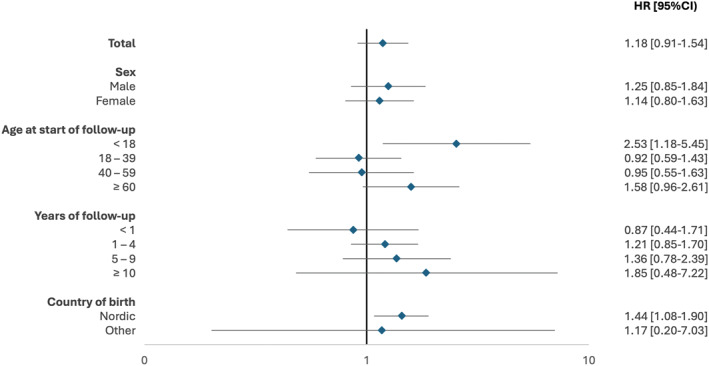
Forest plot of hazard ratios (HRs) of autoimmune thyroid disease (AITD) in patients diagnosed with eosinophilic esophagitis (EoE) compared to general‐population comparators, by subgroup. CI, Confidence interval; HR, Hazard Ratio.

Stratified analyses revealed that age had a significant influence on the results. Patients diagnosed with EoE in childhood (age < 18 years) had an increased risk of future AITD with an HR of 2.53 (95% CI, 1.18–5.45; number of AITD events [EoE/Reference], 10/19). This risk increase dissipated in patients diagnosed after the age of 18 (number of AITD events [EoE/Reference], 60/252; Figure 2). There was also a suggested trend of increasing HR correlating to years of follow‐up, although none of the specific subgroups had a statistically significant HR. Sex or country of birth did not influence the risk estimates as compared to the overall risk estimate.

#### 3.2.2 Sensitivity Analyses

A number of sensitivity analyses were performed, including restricting EoE patients to those diagnosed in 2012 or later, as specified in the Method section. However, all of these analyses yielded similar risk estimates as in the main analysis (Supporting Information S1: Table S3). Notably, the number of AITD events since 2012 was 61 for EoE patients as compared with 239 in reference individuals.

### 3.3 Risk of Prior Autoimmune Thyroid Disease

In the case‐control analysis, 6061 patients with EoE were assessed. The prevalence of prior AITD in patients diagnosed with EoE was 3.9% (*n* = 260) as compared with 3.0% (*n* = 1016) in the general‐population controls, corresponding to an OR of 1.29 (95% CI, 1.10–1.49; *p* = 0.001; Table 4). Hypothyroidism accounted for more than 97% of AITD cases in patients with EoE (230 out of 235) as well as in controls (856 out of 885).

**TABLE 4:** Prior autoimmune thyroid disease (AITD) in patient diagnosed with eosinophilic esophagitis (EoE) as compared with general‐population comparators (reference).

	Reference^a^ (*n* = 29,434)	EoE (*n* = 6061)
Number of events	885	235
Prevalence proportion, %	3.01	3.88
Odds ratio [95% CI]	0 (ref)	1.29 [1.10–1.49]

Abbreviations: CI, Confidence interval; EoE, Eosinophilic esophagitis.

^a^ General‐population references were matched on age, sex, county of residence, and calendar year of diagnosis.

#### 3.3.1 Stratum‐Specific Analyses

**FIGURE 3:**
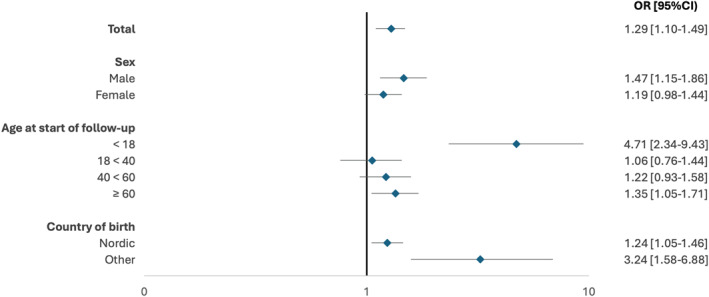
Forest plot of odds ratios (ORs) of prior autoimmune thyroid disease (AITD) in patients diagnosed with eosinophilic esophagitis (EoE) compared to general‐population comparators, by subgroup. CI, Confidence interval; OR, Odds Ratio.

As observed in the cohort analysis, the association between EoE and prior AITD was strongest for patients diagnosed with EoE in childhood who had an OR of 4.71 (95% CI, 2.34–9,43), whereas those diagnosed with EoE after the age of 18 had similar odds of prior AITD as overall (Figure 3). Furthermore, and in line with the cohort analysis, sex or country of birth did not significantly affect the risk estimates compared with the overall risk estimate.

## 4 Discussion

In this population‐based matched cohort study, we found that patients diagnosed with EoE did not have an increased risk of future AITD. However, and in line with previous studies [9, 10], there were increased odds of prior AITD at the time of EoE diagnosis. By examining the relationship in more detail, we were also able to reveal that this association was mainly driven by childhood‐onset EoE, which was associated with both prior and future AITD. Of note, these results do not necessarily suggest a causation, but merely represent an association of these two conditions.

Our prevalence of AITD in patients with EoE (3.9%) was slightly higher than previously reported (0.8%–1.2%) [9, 10]. However, compared to the general population, the OR of prior AITD was in fact lower in our study (1.29) compared to what has previously been reported (3.09) [10]. Notably, the previous study [10], based on the Utah Population Database, had relatively few cases of AITD among patients with EoE (36/4374; 0.8%), and more importantly, even fewer cases of AITD among controls (86/243,236, 0.04%). A possible reason for this discrepancy could be that the study relied on ICD codes alone to identify AITD, leading to exceptionally low rates of AITD in the comparison group, whereas we also took advantage of data on dispensed medications to capture AITD. Indeed, in our study, the vast majority of AITD cases (> 80%) were identified through dispensed medications indicative of AITD. Nonetheless, both studies clearly support the association of EoE and prior AITD.

Among the mechanisms behind the association of EoE and prior AITD could be a shared immune‐mediated pathophysiology. Supporting this, prior epidemiological studies have already established associations between AITD and other immune‐mediated diseases, for example celiac disease [23, 24], which has also been linked with EoE [25].

Possible reasons for the unidirectional association observed between EoE and AITD across all ages include differences in the peak age of disease onset as autoimmune hypothyroidism, which accounted for the majority of the AITD events, typically occurs at ages 45–55 years [26]. Given that this cohort had a mean age of 40 at the time of EoE diagnosis, with a mean follow‐up time of around 5 years, a longer follow‐up time may have been required to capture AITD occurring later in life.

Delayed diagnosis of EoE may also have influenced the direction of the relationship by shifting AITD occurrence from *after* to *before* EoE diagnosis [27]. Prior studies have estimated the median delay of EoE diagnosis to more than 6 years [27, 28]. The risk of delayed diagnosis may especially hold true for patients diagnosed with EoE later in life who may have had a milder phenotype of undiagnosed EoE for a longer period of time.

The increased association observed in childhood‐onset EoE is not unique for AITD but has also been observed for studies on EoE and celiac disease (ref). A plausible explanation for the pronounced association between childhood‐onset EoE and AITD could relate to a higher underlying inflammatory activity of the disease. This would be consistent with observations made for childhood‐onset inflammatory bowel disease, which tends to have a more severe disease course than the adult‐onset subtype [29, 30]. As there is currently no evidence supporting a more severe phenotype in childhood‐onset EoE, this is merely a speculative hypothesis. A more pragmatic explanation for the increased risk of AITD in children with EoE could instead be the more comprehensive follow‐up provided within pediatric care leading to improved case‐finding of subclinical AITD. Nevertheless, it is an observation well worth noting, and it reinforces the importance of subgroup analyses based on age at disease onset in future studies of EoE.

From a clinical perspective, it is important to emphasize that even though EoE may not generally infer an excess risk of developing AITD, these conditions are still likely to coexist. This coexistence is indeed relevant for physicians to consider as EoE and AITD are known to share several symptoms (e.g., fatigue and depression) and subclinical hypothyroidism could be a potential contributor to the increased fatigue observed in patients with EoE [31]. Furthermore, the anatomical proximity of the thyroid and the esophagus could lead patients with known AITD to possibly misattribute symptoms of EoE to their underlying thyroid disease, although this may be less likely.

Strengths of this study include that to our knowledge, it is the first to specifically examine the temporal relationship between EoE and AITD using a nationwide population‐based cohort, thus limiting the risk for selection bias. The dual study design (cohort and case‐control) also enabled us to study both the risk of *prior* and *future* AITD among patients with incident EoE. We also used a previously validated case‐finding strategy for biopsy‐proven EoE (PPV, 89%) [18], in combination with ICD codes recorded in the National Patient Register, which has been shown to have a high diagnostic accuracy (PPV, 80%–95%) [12, 32]. This ensured a high specificity of EoE in the cohort, even though misclassifications may still have occurred. Furthermore, similar to other epidemiological studies investigating thyroid dysfunction [20, 21], we used ATC codes recorded in the Prescribed Drug Register, which has a complete nationwide coverage of dispensed prescriptions since 2005, to identify AITD managed in primary healthcare, which increased our sensitivity and ultimately limited the risk for surveillance bias. However, cases of subclinical hypothyroidism (mildly elevated thyroid‐stimulating hormone but normal thyroid hormones) may still be missed as they may not always receive medical treatment. Finally, an important strength of this study was our use of sibling comparators as they allowed us to control for shared genetics and early‐life environmental factors.

Limitations of the study include a limited number of future AITD events, which makes interpretation of the cohort analysis more challenging. Although our study did not support an association between EoE and future AITD, we cannot exclude a type 2 error. Similarly, we cannot exclude a type 1 error with regard to the association found between childhood‐onset EoE and AITD, although the presence of a similar age‐specific pattern for other immune‐mediated comorbidities of EoE, such as celiac disease, strengthens the suspicion of a true association [25]. Other limitations include the lack of access to primary healthcare records, which would have allowed us to reconfirm AITD occurrence and more accurately define the date of AITD onset, as well as the lack of data on clinical characteristics of patients with EoE. Additionally, we did not have enough statistical power to investigate the risk of AITD by subtype (hypo‐ or hyperthyroidism) due to the low number of hyperthyroidism events. It is also possible, though unlikely, that the study was underpowered to detect such small differences in risk for future AITD despite more than 30,000 PY in almost 6000 patients with EoE. Furthermore, since EoE remains relatively recently acknowledged, most patients had less than 5 years of follow‐up, which limited our possibilities to investigate the long‐term risk of AITD. Therefore, future studies may be needed to examine the long‐term risk of AITD in patients with EoE when even longer follow‐up is available. We also acknowledge that there is always a risk of residual confounding despite our efforts to adjust for covariates believed to be associated with the exposure and the outcome. Similarly, it is important to emphasize that as with all observational studies, we cannot draw any conclusions on the causal relationship between these diseases.

In conclusion, although patients with EoE do not generally seem to be at an increased risk of *future* AITD, the odds of *prior* AITD were increased at the time of EoE diagnosis. Furthermore, childhood‐onset EoE was associated with both *prior* and *future* AITD. Given the increased coexistence of AITD in patients with EoE, physicians diagnosing EoE should be observant of symptoms indicative of AITD.

## Author Contributions

ICMJE criteria for authorship read and met: All authors. Agree with the manuscript's results and conclusions: All authors. Designed the study: S.R.B., J.F.L. Collected data and responsible for data integrity: J.F.L. Analyzed the data: S.R.B., B.R. Wrote the first draft of the paper: S.R.B. Contributed to the writing of the paper: All authors. Interpretation of data: All authors approved the final version of the manuscript. Supervised the project: J.F.L. Obtained funding: S.R.B., J.F.L. Guarantor of the article: S.R.B., J.F.L.

## Funding

This study was supported by the Karolinska Institute and *Magtarmfonden*.

## Ethics Statement

The study was approved by the Regional Ethical Review Board in Stockholm (reference number: 2014/1287–31/4, 2017/1497–32 and 2022–05774‐02).

## Consent

Informed consent was waived since the study was strictly register‐based.

## Conflicts of Interest

S.R.B. has received consultant fees from Takeda, unrelated to this work. J.F.L. has coordinated a study on behalf of the Swedish IBD quality register (SWIBREG), which received funding from Janssen corporation. J.F.L. has also received financial support from MSD developing a paper reviewing national healthcare registers in China and for an unrelated study on IBD. J.F.L. also has an ongoing research collaboration with Takeda about celiac disease and chronic liver disease with this company. None of this funding is related to this work. M.C. has received speaker’s fees from ViforPharma and AbbVie. She is the national PI for clinical trials for AstraZeneca. A.M.U. is a medical advisor and/or consultant for Takeda, Sanofi‐Regeneron, Uniquity, and Areteia. None of these activities are related to this work. The other authors report no conflicts of interest.

## Supporting information


Supporting Information S1

## Data Availability

The data that support the findings of this study are available on request from the corresponding author. The data are not publicly available due to privacy or ethical restrictions.
