# Supplementary material for: Eosinophilic Esophagitis and Autoimmune Thyroid Disease: A Population‐Based Matched Cohort Study
Source: United European Gastroenterol J. 2026 Jul 1;14(6):e70248. doi: 10.1002/ueg2.70248 (PMC13320827; doi:10.1002/ueg2.70248)
Supplement: Supplementary file 1 — Supporting Information S1 [file UEG2-14-e70248-s001.docx]

**Eosinophilic esophagitis and autoimmune thyroid disease:
a population-based matched cohort study**

**SUPPLEMENTAL MATERIAL**

**Authors’ affiliations**

Soran R. Bozorg, MD, PhD, BSc ^1,2^, David Bergman, MD, PhD ^1^, Fahim Ebrahimi, MD, MSc ^1,3^, Bjorn Roelstraete, PhD ^1^, Marie Carlson, MD, PhD ^4^, Amiko M. Uchida, MD ^5^, Evan S. Dellon, MD, MPH ^6^, and Jonas F. Ludvigsson, MD, PhD ^1,7,8^

1 Department of Medical Epidemiology and Biostatistics, Karolinska Institutet, Sweden

2 Division of Gastroenterology, Karolinska University Hospital, Sweden

3 Department of Gastroenterology, Clarunis University Centre for Gastrointestinal and Liver Disease, Switzerland

4 Department of Medical Sciences, Uppsala University, Sweden

5 Division of Gastroenterology, Hepatology, & Nutrition, University of Utah School of Medicine, UT, USA

6 Department of Medicine, University of North Carolina School of Medicine, NC, USA

7 Department of Pediatrics, Örebro University Hospital, Sweden

8 Department of Medicine, Columbia University Medical Center, NY, USA

**Correspondence**:

Soran Rabin Bozorg, MD PhD BSc

Department of Medical Epidemiology and Biostatistics, Karolinska Institutet, Sweden

SE-171 77 Stockholm, Sweden

Phone: 08-524 80000

E-mail: soranbozorg@gmail.com

**SUPPLEMENTAL TABLES AND FIGURES**

**Supplemental Figure 1.** Flowchart of study population.

**Supplemental Table 1.** Definitions of outcomes, exposures, and comorbidities according to International Classification of Diseases codes, the Anatomical Therapeutic Chemical codes, or Swedish procedure codes (Swedish: KVÅ-koder).

**Supplemental Table 2.** Baseline characteristics of patients diagnosed with eosinophilic esophagitis and sibling comparators.

**Supplemental Table 3.** Sensitivity analyses of the risk of autoimmune thyroid disease (AITD) in patients diagnosed with eosinophilic esophagitis (EoE) compared with general-population comparators.

**Supplemental Figure 1.** Flowchart of study population.


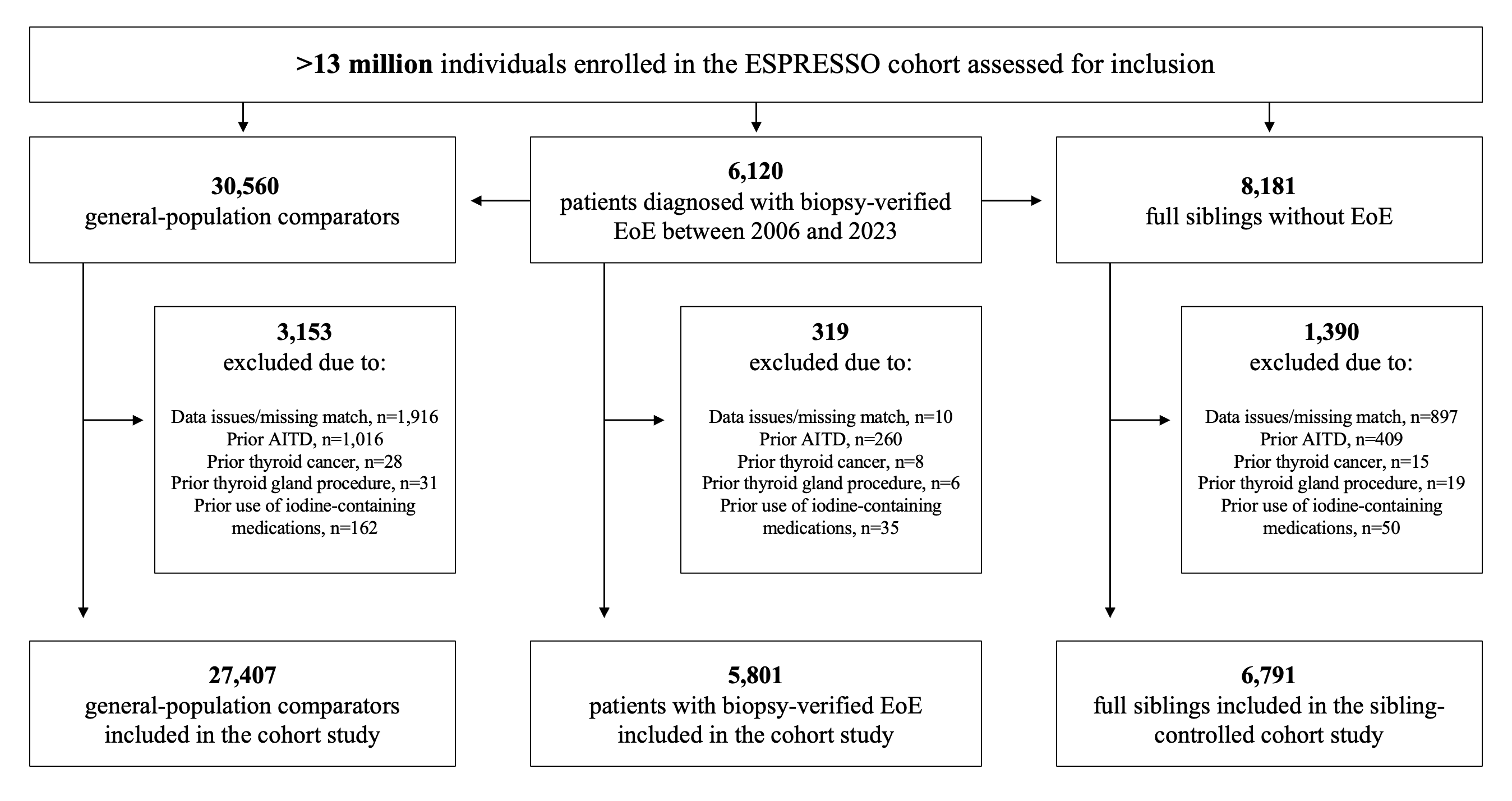


Abbreviations: AITD, Autoimmune thyroid disease; EoE, Eosinophilic esophagitis; ESPRESSO, Epidemiology Strengthened by histoPathology Reports in Sweden.

**Supplemental Table 1.** Definitions of outcomes, exposures, and comorbidities according to International Classification of Diseases (ICD) codes, the Anatomical Therapeutic Chemical (ATC) codes, or Swedish procedure codes (Swedish: KVÅ-koder).

|  |  |  |  |
| --- | --- | --- | --- |
| **Disease, drug group, or procedure** | **ICD-10/ICD-9** | **ATC** | **Procedure code** |
|  |  |  |  |
|  |  |  |  |
| **Eosinophilic esophagitis** | K20.9A/- |  |  |
| **Autoimmune thyroid disease** Hashimoto’s disease Grave’s disease Grave’s ophthalmopathy | E06.3/245.2  E05.0/242.0  H06.2/376.4 |  |  |
| **Thyroid cancer** | C73/193 |  |  |
| **Thyroid hormone substitution**  Levothyroxine, T4  Liothyronine, T3 |  | H03AA01  H03AA02 |  |
| **Iodine-containing drugs**  Amiodarone  Lithium  Interferon alfa  Interferon alfa 2a  Interferon alfa 2b |  | C01BD01  N05AN01  L03AB01  L03AB04  L03AB05 |  |
| **Thyroid gland procedures** |  |  | BAA05, BAA1x, BAA2x, BAA3x, BAA4x, BAA5x, BAA6x, BAA9 |
| **Concomitant comorbidities**  Atopy  *Asthma*  *Allergic rhinitis*  *Atopic eczema*  Celiac disease  Inflammatory bowel disease  Chronic obstructive pulmonary disease (COPD) ^ | J46-46/493  J30/477  L20/691  K90.0/579.0  K50-51/555-556  J41-44/491-92, 496 |  |  |
|  |  |  |  |

Abbreviations: ATC, Anatomical Therapeutic Chemical; ICD, International Classification of Diseases.
^ Restricted to those diagnosed at age 40 or later.

**Supplemental Table 2.** Baseline characteristics of patients diagnosed with eosinophilic esophagitis (EoE) and sibling comparators (reference).

Abbreviations: EoE, Eosinophilic esophagitis; IQR, Interquartile range; SD, Standard deviation.
^ Defined as asthma, allergic rhinitis, or atopic eczema (see Suppl. Table 1).
° Restricted to those diagnosed at age 40 or later.

|  | **EoE (n=4253)** | **Reference (n=6791)** |
| --- | --- | --- |
|  | n [%] | n [%] |
| **Sex** |  |  |
| Male | 3182 [74.82] | 3582 [52.75] |
| Female | 1071 [25.18] | 3209 [47.25] |
| **Age at start of follow-up** |  |  |
| Mean [SD] | 38.64 [19.42] | 39.43 [19.94] |
| Median [IQR] | 39.00 [23.00-53.00] | 40.00 [24.00-55.00] |
| Range, min-max | 0-86 | 0-88 |
| < 18 | 796 [18.72] | 1171 [17.24] |
| 18 – 39 | 1339 [31.48] | 2154 [31.72] |
| 40 – 59 | 1464 [34.42] | 2258 [33.25] |
| ≥ 60 | 654 [15.38] | 1208 [17.79] |
| **Country of birth** |  |  |
| Nordic countries | 4147 [97.51] | 6516 [95.95] |
| Other | 106 [2.49] | 275 [4.05] |
| **Highest attained education level** |  |  |
| Compulsory school (≤ 9 years) | 541 [12.72] | 840 [12.37] |
| Upper secondary school (10 – 12 years) | 1491 [35.06] | 2357 [34.71] |
| College or university (≥ 13 years) | 1414 [33.25] | 2245 [33.06] |
| Missing | 807 [18.97] | 1349 [19.86] |
| **Comorbidity at start of follow-up** |  |  |
| Atopy ^ | 1184 [27.84] | 992 [14.61] |
| Chronic pulmonary obstructive disease (COPD) ° | 25 [0.59] | 52 [0.77] |
| Inflammatory bowel disease (IBD) | 114 [2.68] | 73 [1.07] |
| Celiac disease | 103 [2.42] | 54 [0.80] |
| **Calendar year at start of follow-up** |  |  |
| 2006 – 2012 | 473 [11.12] | 806 [11.87] |
| 2013 – 2017 | 1353 [31.81] | 2190 [32.25] |
| 2018 – 2023 | 2427 [57.07] | 3795 [55.88] |
| **Years of follow-up** |  |  |
| Mean [SD] | 5.78 [3.69] | 5.85 [3.71] |
| Median [IQR] | 5.03 [2.85-8.27] | 5.09 [2.89-8.47] |
| Range, min-max | 0.01-17.88 | 0.00-17.68 |
| < 1 | 231 [5.43] | 380 [5.60] |
| 1 – 4 | 1895 [44.56] | 2971 [43.75] |
| 5 – 9 | 1486 [34.94] | 2363 [34.80] |
| ≥ 10 | 641 [15.07] | 1077 [15.86] |
| **Censoring events during follow-up** |  |  |
| Thyroid cancer | 0 [0.00] | 3 [0.04] |
| Use of iodine-containing drug | 16 [0.38] | 24 [0.36] |
| Thyroid gland procedure | 0 [0.00] | 0 [0.00] |

**Supplemental Table 3.** Sensitivity analyses of the risk of autoimmune thyroid disease (AITD) in patients diagnosed with eosinophilic esophagitis (EoE) compared with general-population comparators (reference).

|  |  |  | **Reference *** |  |  |  | **EoE** |  |  | **Hazard ratio (baseline model)** |
| --- | --- | --- | --- | --- | --- | --- | --- | --- | --- | --- |
|  |  | N | Events | Incidence rate per 1000 person-years [95%CI] |  | N | Events | Incidence rate per 1000 person-years  [95%CI] |  | [95%CI] |
|  |  |  |  |  |  |  |  |  |  |  |
| Restricting study population to EoE patients identified by SNOMED code M4715 (*eosinophilic inflammation*) |  | 24218 | 244 | 1.75 [1.54-1.99] |  | 5112 | 62 | 2.12 [1.62-2.71] |  | 1.16 [0.88-1.54] |
|  |  |  |  |  |  |  |  |  |  |  |
| Restricting start of follow-up to ≥ 2012 |  | 25446 | 239 | 1.84 [1.62-2.09] |  | 5396 | 61 | 2.23 [1.71-2.86] |  | 1.17 [0.88-1.55] |
|  |  |  |  |  |  |  |  |  |  |  |
| Restricting outcome occurrence to AITD identified by ICD codes |  | 26829 | 45 | 0.30 [0.22-0.40] |  | 5729 | 11 | 0.34 [0.17-0.61] |  | 1.10 [0.57-2.13] |
|  |  |  |  |  |  |  |  |  |  |  |
| Excluding first year of follow-up |  | 25735 | 219 | 1.42 [1.24-1.62] |  | 5461 | 60 | 1.84 [1.41-2.37] |  | 1.26 [0.95-1.68] |
|  |  |  |  |  |  |  |  |  |  |  |
| Excluding those without healthcare contacts 6-36 months before diagnosis |  | 13682 | 169 | 2.20 [1.88-2.56] |  | 4138 | 51 | 2.20 [1.64-2.89] |  | 1.01 [0.74-1.38] |
|  |  |  |  |  |  |  |  |  |  |  |

Abbreviations: AITD, Autoimmune thyroid disease; CI, Confidence interval; EoE, Eosinophilic esophagitis; HR, Hazard ratio; ICD, International Classification of Diseases; SNOMED, Systematized Nomenclature of Medicine. * General-population references were matched on age, sex, county of residence, and calendar year of diagnosis.
